# Medication Burden Before and After Prescription of Biologics in Patients with Inflammatory Bowel Disease

**DOI:** 10.3390/jcm13216408

**Published:** 2024-10-25

**Authors:** Annika Fernandez Milano, Sarah Krieg, Karel Kostev

**Affiliations:** 1 Epidemiology, IQVIA, 60549 Frankfurt, Germany; 2 Department of Inclusive Medicine, University Hospital Ostwestfalen-Lippe, Bielefeld University, 33617 Bielefeld, Germany; 3 University Clinic, Philipps-University, 35043 Marburg, Germany

**Keywords:** inflammatory bowel disease, Crohn’s disease, ulcerative colitis, biologics, medication burden

## Abstract

**Background:** Biologics are a cornerstone in the treatment of severe cases of inflammatory bowel disease (IBD) and aim to control the disease and improve quality of life. This study investigated changes in nonbiologic medication prescriptions for IBD patients initiating biologic therapy in Germany. **Methods:** This study used data from anonymized pharmacy records in the German longitudinal prescription (LRx) database and included biologic-naive IBD patients who received their first biologic therapy prescription between 2016 and 2022. Changes in prescription rates and pill counts for nonbiologic medications (corticosteroids, 5-aminosalicylates (5-ASA), proton pump inhibitors, analgesics, immunosuppressants, Vitamin D, iron, and antibiotics) before and after the initiation of biologic therapy were assessed using descriptive statistics, McNemar’s tests, and Poisson regression models, adjusting for age and sex. **Results:** A total of 29,559 biologic-naive IBD patients were included. Prior to index, 91.2% received at least one nonbiologic medication prescription, where corticosteroids and 5-ASA were the most common. Postindex, the overall prescription rate decreased to 87.7%, with significant reductions in prescriptions observed for corticosteroids, 5-ASA, and immunosuppressants (*p*-values < 0.001). The mean (SD) pill count dropped from 704 (1712) to 514 (1651), with the largest mean differences (95% CI) having been for corticosteroids (−77.9 [−80.3 to −75.5]), 5-ASA (−61.6 [−65.2 to −58.1]), and immunosuppressants (−55.0 [−57.5 to −52.6]). Older patients tended to have greater decreases in pill counts for corticosteroids and 5-ASA, while males showed statistically significant reductions in pill count for immunosuppressants compared with females. **Conclusions:** This study demonstrates that the prescription of nonbiologic medications significantly decreased after biologic therapy initiation. The use of biologics may therefore lead to improved disease management and potentially better patient outcomes.

## 1. Introduction

The chronic inflammatory bowel diseases (IBD), Crohn’s disease (CD) and ulcerative colitis (UC), are immune-mediated diseases primarily of the gastrointestinal tract for which there is no cure [1]. The diseases’ relapsing but chronically active course [2] are primarily characterized by abdominal pain, diarrhea, and rectal bleeding [3,4]. This can be physically, psychosocially, and professionally very limiting for individuals while also placing a financial burden on the healthcare system and the economy as a whole [5,6,7,8]. Worldwide, approximately 4.9 million people suffered from IBD in 2019. In Germany, it is estimated that between 320,000 and 470,000 people are affected [9,10]. Around 30% of patients experience a continuous disease course with alternating phases of relapse and remission [11,12].

Despite the unexplained pathogenesis, genetic predisposition, as well as environmental factors, are presumed to be responsible for the disease’s occurrence through a disruption of the epithelial barrier function and reduced diversity of the microbiome. Risk factors are thought to include an inflammation-promoting diet, stress, smoking in CD, and medications such as antibiotics [11,13,14].

The therapies used aim primarily at controlling the disease and improving the quality of life (QoL) [11,15]. The treatment regime depends on the disease phase (relapse or remission), severity of symptoms, patient age (child or adult) and sex, and the drug’s side effect profile [16,17]. Corticosteroids or aminosalicylates (5-ASA) are conventionally used to induce remission for the first time. In cases of steroid-refractory courses, but also to maintain remission, patients are often switched to immunosuppressants (e.g., azathioprine or methotrexate) [12,16,18]. However, around a third of patients must discontinue this therapy because of serious side effects [19]. Therefore, therapy escalation with biologics, more cost-effective biosimilars, or targeted therapies (small molecules) is indicated in cases of treatment failure or particularly severe disease progression, which affects around 40–50% of patients [12,16].

The first biologics to be approved in Germany were the tumor necrosis factor (TNF)-alpha inhibitors infliximab, adalimumab, and golimumab (for UC only). While the integrin inhibitor vedolizumab and the interleukin (IL)-12/23 inhibitor ustekinumab have better side effect profiles, infliximab is the most effective compared with other biologics [11,12,18]. The other biologics that were approved in 2022 and 2023 include the IL-23 inhibitors risankizumab (for CD only) and mirikizumab (for UC only) [11,20]. Since their safety profile still remains unknown, the newer targeted therapies, which include the Janus kinase (JAK) inhibitors filgotinib (for UC only) and upadacitinib and the sphingosine-1-phosphate receptor (S1PR) ozanimod (for UC only), are used mainly when the biologics have failed [11,12,16].

Adherence is crucial for the success of therapy and appears to be better with biologic therapies than with conventional therapies [21]. In addition, biologic therapies are reported to improve treatment outcomes by reducing hospitalization rates, increasing patient satisfaction, and causing fewer side effects [22,23]. However, in practice, biologic therapies are prescribed less frequently than recommended by the guidelines [9,12]. Furthermore, real-world studies are important, as demographic or clinical characteristics of patients in the real-world care may differ from participants of clinical trials [24].

Given that biologics may alleviate symptoms and have fewer side effects, a reduction in the use of other medications seems reasonable. However, there is limited evidence regarding changes in the use of other medications before and after the initiation of biologic therapy in IBD treatment in Germany outside of clinical trials. One study compared the costs of continuous biologic use with nonuse in Germany [25], while another assessed the use and costs of steroids and immunosuppressants before and after biologic therapy in patients with UC [26]. In light of the importance of symptom control in improving QoL, this study aimed to investigate changes in nonbiologic prescriptions among IBD patients, encompassing both UC and CD, after the introduction of biologic therapy, using real-world data from Germany.

## 2. Data Source and Methods

### 2.1. Data Source

Data from the German longitudinal prescription (LRx) database, a national database (IQVIA) that includes prescription data of patients with statutory health insurance (SHI) from retail pharmacies, were used for this study. The database contains demographic data (e.g., age, sex) and product information for each prescription (e.g., brand, substance, package size, product form, number of packages, issue date). To ensure data privacy, the information was encrypted before transmission to IQVIA. While diagnoses and laboratory data were not available, patients’ historical data across medical specialties and health conditions were collected. The LRx database represents around 80% of all prescriptions that are processed for SHI reimbursement [27]. Multiple studies, including analyses on IBD, have been conducted with the database before [28]. Because of the anonymized nature of the data, no institutional review board approval or informed consent was required as per German law for studies using this secondary database.

### 2.2. Study Population

**Figure 1:**
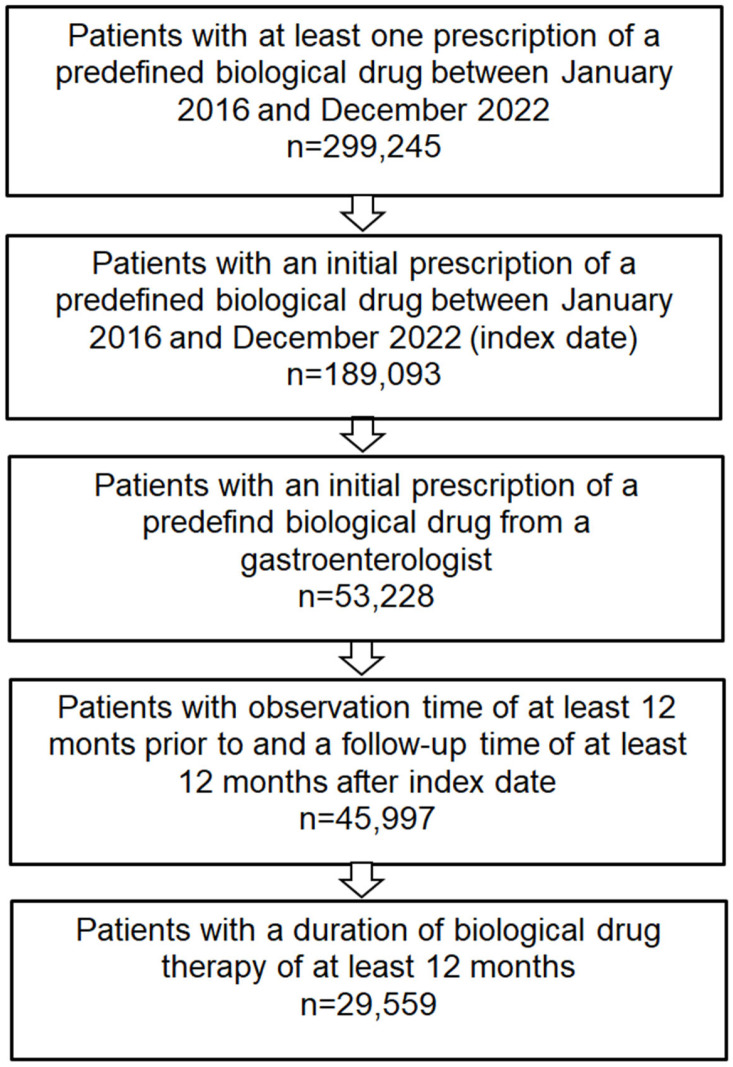
Selection of study population.

The patient selection process is displayed in Figure 1. All patients irrespective of age, who received a biologic therapy prescription (infliximab, adalimumab, golimumab, vedolizumab, or ustekinumab) between 1 January 2016 and 31 December 2022 from a gastroenterologist were selected from the LRx database for analysis. The date of the first prescription was used as the index date. Patients had to be observable in the database at least 12 months prior to and post the index date and were required to be on biologic therapy for at least 12 months.

### 2.3. Study Outcomes

The outcomes were the prescription rates and the number of prescribed pills for each nonbiologic medication, which were compared for the 12 months prior to and after index. Only nonbiologic medications that were prescribed to at least 10% of all patients were considered, and they were grouped as follows (with corresponding EphMRA codes if applicable): corticosteroids (H02A, A07E2, M01B), 5-ASA (A07E1), proton pump inhibitors (PPIs) (A02B2), analgesics (N02B, M01A), immunosuppressants other than biologics (L04X except for infliximab, adalimumab, golimumab, vedolizumab, or ustekinumab), Vitamin D, iron, antibiotics (J01). Most of these medications are also used in clinical practice or recommended by treatment guidelines for IBD patients [2,3,4].

The number of pills was estimated as a product of the package count and size. To reduce the influence of outliers, pill counts above the 99th percentile were capped. For each medication, patients without prescriptions were assigned values of 0 in the analyses.

### 2.4. Statistical Analyses

Categorical variables were described using counts and percentages, while continuous variables were described using counts, means, and standard deviations. To assess changes in the number of patients on each medication type before and after the index date, McNemar’s tests, specifically for paired data, were conducted. Poisson regression models were used to compare pill counts before and after biologic therapy, adjusting for age and sex while correcting for overdispersion and controlling for repeated measures within patients. For key medications, the difference in pill count between the two periods was further stratified by age group and sex using Poisson regression models. The stratified models by age were adjusted for sex, and the stratified models by sex were adjusted for age.

## 3. Results

### 3.1. Patient Characteristics

Of the original 299,245 patients who received at least one prescription of a biologic, 29,559 patients were selected for analysis. This resulted in a biologic persistence rate of 64.3%. The selected patients had a mean (SD) age of 41.1 (15.6) years (Figure 1 and Table 1). Most patients (53.3%) were aged 40 years or younger, and a similar proportion of patients were female (48.0%) and male (46.3%). Between 2016 and 2022, the number of patients initiating biologic therapy each year increased, with the lowest proportion of patients starting in 2016 (9.9%) and the highest in 2021 (18.1%). The most frequent drug was adalimumab (36.9%), followed by infliximab (33.7%) and vedolizumab (19.8%).

### 3.2. Medication Prescriptions

In the 12 months preceding the index date, 91.2% of patients were prescribed at least one of the medications included in the study. Corticosteroids and 5-ASA were the most frequently prescribed medications, with 74.5% and 53.3% of patients receiving these treatments, respectively (Table 2). Analgesics and PPIs were prescribed to similar proportions of patients, at 42.4% and 41.9%, respectively. Approximately one-third of patients (29.5%) were prescribed antibiotics, while immunosuppressants were given to 26.9%. Fewer than 20% of patients received prescriptions for Vitamin D and iron supplements.

In the 12 months following the index date, the overall prescription rate decreased to 87.7%. The proportion of patients receiving prescriptions for all medications, except iron supplements (which saw an increase), significantly declined (all *p*-values < 0.001). The number of patients who were prescribed analgesics and antibiotics remained largely unchanged between the two periods, but the use of immunosuppressants was halved (13.0%), and prescriptions for corticosteroids and 5-ASA dropped by a third (to 52.7% and 36.5%, respectively). The proportion of patients prescribed PPIs also fell to 31.2%.

### 3.3. Medication Pill Count

The overall adjusted mean difference (95% CI) in the pill count from the pre- to the postindex period was −193.5 (−199.8 to −187.2), dropping from a mean (SD) number of prescribed pills of 704 (1712) to 514 (1651) (Table 3). Despite most medications showing a statistically significant decrease (*p*-values < 0.001), the adjusted mean difference (95% CI) was highest for corticosteroids (−77.9 [−80.3 to −75.5]), 5-ASA (−61.6 [−65.2 to −58.1]), and immunosuppressants (−55.0 [−57.5 to −52.6]). Analgesics, antibiotics, Vitamin D, and iron supplements averaged fewer than 40 prescribed pills per patient in the preindex period. Although the pill counts slightly increased for analgesics and iron supplements, with mean differences (95% CI) of 2.4 (1.5 to 3.2) and 0.8 (0.3 to 1.3), respectively, the number of pills per patient decreased for PPIs and Vitamin D. However, this decrease was less pronounced, with mean differences (95% CI) of −6.6 (−9.5 to −3.7) and −1.5 (−2.1 to −0.8), respectively. Antibiotics were the only medication for which the mean difference was not statistically significant (*p*-value ≥ 0.050).

### 3.4. Medication Pill Count by Age and Sex

Since the most significant pill count changes between the pre- and postindex periods occurred with corticosteroids, 5-ASA, and immunosuppressants, these were subsequently stratified by age and sex (Table 4). All sex-adjusted mean differences within each age group, as well as age-adjusted mean differences within each sex group, were statistically significant (*p*-values < 0.001).

Pill counts for corticosteroids and 5-ASA increased with age in both periods, but the mean differences between pre- and postindex periods were also larger in older patients. For instance, patients aged ≤ 30 years had a mean (SD) corticosteroid pill count of 169 (183) before and 92 (159) after the index, whereas those aged >60 years averaged 213 (SD: 225) before and 129 (SD: 197) after the index. The mean difference (95% CI) was highest for those >60 years and lowest for the 31 to 40 age group, with −84.4 (−91.6 to −77.3) and −73.7 (−78.8 to −68.7), respectively. The mean difference (95% CI) in pill count of 5-ASA was also highest for patients over 60 years old and lowest for those aged 31 to 40 years, with values of −66.6 (−77.6 to −55.6) and −56.0 (−63.3 to −48.8), respectively. Despite these tendencies, the confidence intervals for the mean differences overlapped between the age groups. The observed trends did not apply to the number of prescribed pills for immunosuppressants. In this case, the 41- to 50-year-olds had the highest mean difference (95% CI), while the oldest age group (over 60 years) showed the lowest mean difference (95% CI), with values of −62.7 (−69.1 to −56.3) and −48.0 (−54.1 to −41.9), respectively.

In general, the pill count was lower for females than for males in both the pre- and postindex periods across the medications studied. For example, female patients averaged 233 (SD: 372) and 171 (SD: 348) prescribed pills of 5-ASA in the pre- and postindex periods, respectively, while males averaged 253 (SD: 378) and 190 (SD: 358), respectively. However, for corticosteroids and 5-ASA, the mean differences (95% CI) in pill count were similar between both groups. The most significant sex difference in the mean change (95% CI) in pill count was observed for immunosuppressants, with −51.4 (−54.6 to −48.2) for females versus −60.2 (−64.1 to −56.3) for males.

## 4. Discussion

This study demonstrated a reduction in the overall medication burden for patients on biologic therapy from the pre- to the postindex period. Corticosteroids and 5-ASA remained the most frequently prescribed medications across both periods. Significant reductions were observed in the use of these medications, as well as in immunosuppressants. Additionally, moderate decreases were seen in PPI prescriptions, while iron supplementation slightly increased. Minimal changes were noted in the prescriptions for antibiotics, Vitamin D supplements, and analgesics. These patterns were similarly observed in the pill counts, with older patients showing a tendency towards greater reductions in corticosteroids and 5-ASA, while males had statistically significant reductions in immunosuppressants compared with females.

The demographic characteristics of our study population were consistent with those reported in other studies of IBD patients, which focused primarily on costs and healthcare resource utilization related to biologic therapy based on data from German statutory health insurance claims [25,26,29]. Each of these studies primarily involved middle-aged IBD patients and demonstrated a similar sex distribution. After 12 months, our study revealed a biologic therapy persistence rate of 64.3%, while the study by Mahlich et al. reported a slightly higher rate of 72.2% [25].

Corticosteroid prescription rates in our study were 74.5% in the 12-month preindex period, aligning with other studies that reported rates ranging from 64.1% to 84.2% [25,26,29]. In our study, there was a significant reduction in corticosteroid prescription postindex, supporting the steroid-sparing effect of biologic therapies. This finding is consistent with the study by Dignass et al. on UC patients, which also showed a decrease in corticosteroid use after 12 months of biologic therapy, although at a higher postindex rate of 64.8% compared with 52.7% in our study [26]. The overall reduction in corticosteroid use following biologic therapy is clinically important, given the known risks associated with chronic corticosteroid use, such as infections, osteoporosis, and adrenal suppression. Because of these side effects, long-term corticosteroid treatment, while effective for inducing remission, should be avoided [30]. However, pretreatment with corticosteroids may also help reduce the risk of antidrug antibody (ADA) formation caused by biologics, as demonstrated with intravenous (IV) corticosteroids in CD patients treated with infliximab [31]. To assess the response to corticosteroids and determine the optimal timing for discontinuation in biologic-treated patients, noninvasive biomarkers such as fecal calprotectin and myeloperoxidase may be valuable tools [32].

The prescription rate for the anti-inflammatory agent 5-ASA decreased from 53.3% to 36.5% in our study population, indicating the effectiveness of biologic therapy. Other studies investigating the benefit of concomitant use of 5-ASA with biologics showed higher prescription rates at the induction of biologic therapy [33,34]. Singh et al. conducted a pooled analysis of trial data, where prescription rates ranged from 45.5% to 83.6%, with an average of 78.6% across all patients. This study found that the concomitant use of 5-ASA in UC patients treated with infliximab and golimumab was not associated with achieving clinical remission, neither during induction nor maintenance of biologic therapy [33]. Another study by Choi et al. assessed the efficacy of concomitant 5-ASA use in terms of event-free survival for patients starting biologic therapy and found that IBD patients who continued 5-ASA concomitantly did not experience better outcomes than those receiving biologic therapy alone [35].

Notably, although long-term use of 5-ASA may provide chemoprevention against colorectal cancer in UC patients by controlling inflammation [36,37], the European Crohn’s and Colitis Organization (ECCO) guidelines do not support its use as a first-line treatment solely for its chemopreventive effect because of a lack of sufficient evidence [4].

Our study recorded a significantly lower prescription rate for immunosuppressants, at 26.9% preindex, compared with other studies, which reported rates ranging from 53.1% to 58.9% [25,26,29]. Postindex, the reduction was even more pronounced, with rates dropping to 13.0%, in contrast to 35.5% in the study by Dignass et al. [26]. The decrease in immunosuppressant use after the initiation of biologic therapy may indicate an improved disease severity within our study population. The discrepancy with other studies could suggest that treatment practices in our population deviated from guidelines recommending combined therapy with thiopurines for agents such as infliximab in patients with a more aggressive disease, or it may indicate that our patient cohort experienced a milder disease course [26,37]. Additionally, males were prescribed a higher number of pills than females, consistently with findings from a study by Blumenstein et al. in Germany [38], and exhibited a greater reduction in pill count from the pre- to the postindex periods. This difference may be partially attributed to concerns regarding the use of these medications in females of childbearing age [38].

The combination therapy of immunosuppressants and biologics is a well-established strategy in clinical practice because of its favorable therapeutic effects [39,40]. This approach can help reduce immunogenicity, specifically the formation of ADA against biologics, thereby enhancing their effectiveness [41]. However, the increased risk of infections and other adverse events requires careful patient selection and monitoring [42]. Immunosuppressants should be combined with faster-acting drugs, such as corticosteroids or infliximab, in patients with active disease and administered long-term for maintenance rather than episodically [2,43]. Biomarkers such as the HLA-DQA1*05 allele may help identify patients who would benefit from combination therapy [44].

Anemia is one of the most common extraintestinal manifestations in IBD, affecting approximately one-third of IBD patients in Germany [45]. Patients with iron deficiency of anemia (IDA) should receive iron supplementation regardless of disease severity [46,47]. Anti-TNF-alpha agents have been shown to positively influence iron metabolism by controlling the production of proinflammatory cytokines. In patients with anemia of chronic disease (ACD) who experience acute or chronic immune responses [48], the effect of TNF-alpha inhibitors has, however, been found to be independent of iron supplementation [49]. In our study, the iron prescription rate increased from 16.9% to 21.4% postindex, while pill counts remained nearly unchanged at a relatively low level (mean of ≤12 pills per patient). This may have been due to improved health and, consequently, better iron absorption in our study population, as the ECCO guidelines recommend oral iron supplementation only for IBD patients with normal C-reactive protein levels and mild IDA who are in clinical remission. However, patients with severe anemia may have generally received IV iron rather than oral supplements, which would not be reflected in the prescription rate [46,47].

In our study, the prescription rate for PPIs decreased from 41.9% in the pre- to 31.2% in the postindex period. Interestingly, a meta-analysis by Lu et al. found that patients on concurrent PPI and infliximab therapy were less likely to achieve remission [50]. PPIs, which reduce stomach acid production to aid digestion, may negatively affect the effectiveness of biologics by altering the gut microbiota [51]), raising concerns about the concomitant use of PPIs and biologics.

Prescriptions for antibiotics, Vitamin D supplements, and analgesics remained nearly unchanged. Antibiotics may be used to treat infections, maintain remission, manage active luminal disease and perianal fistulas in CD patients, and treat pouchitis in UC patients [52]. The effect of antibiotics on biologic therapy remains unclear [53]. Vitamin D supplementation has been observed to decrease inflammatory cytokines such as TNF-alpha, thereby improving health outcomes [54]. In our study, Vitamin D supplementation levels remained stable, possibly because physicians prescribed it preventatively throughout the disease course or because the need for it was not reduced by biologics. Similarly, biologic therapy did not significantly affect the prescription of analgesics. In general, nonsteroidal anti-inflammatory drugs and opioids should be avoided in patients with IBD because of their potential to exacerbate the disease [55]. However, the overall pill counts for these medications were already relatively low across both periods, with means of ≤40 pills per patient.

## 5. Strengths and Limitations

One significant strength of this study is the large number of patients included in the analysis, which enhances its representativeness of the German IBD population and thus its external validity. To our knowledge, this is also the first study to analyze prescription changes across multiple medication classes used in the treatment of IBD before and after the initiation of biologic therapy in Germany.

The findings should be interpreted in light of several limitations. First, the LRx database did not allow for patient selection by specific diagnoses (e.g., ICD-10 code for UC or CD) or histological data. Although it is probable that individuals receiving these medications in gastroenterology practices had IBD, the inability to further stratify into the CD and UC categories remains an important limitation. Second, the potential initiation of first-line biologic therapy in hospitals, which is not documented in the LRx database, could introduce selection bias. Nevertheless, IBD patients are most commonly treated in an outpatient setting by gastroenterologists, specialist internists, general internists, or general practitioners [6]. Third, the dosage was not considered in the calculation of pill count, which may have influenced the comparison of medication use between the pre- to the postindex periods. Last, individual biological active ingredients could not be analyzed because of the usage policy of the database for noncommercial research projects.

## 6. Conclusions

This study reveals that biologic therapy can significantly reduce the medication burden for IBD patients, particularly in relation to corticosteroids, 5-ASA, and immunosuppressants. The substantial reduction in corticosteroid use underscores the steroid-sparing benefits of biologics and improvements in disease management. However, some patients may still require combination therapies, including immunosuppressants, to minimize immunogenicity and enhance treatment efficacy. Future research should focus on the long-term effects of biologic therapy, differences between UC and CD patients, and the identification of biomarkers to guide more personalized treatment approaches. In addition, a more in-depth analysis on biological active ingredients could provide valuable insight.

## Figures and Tables

**Table 1:** Baseline characteristics of the study population.

Characteristics		N	%
Total		29,559	100%
Age (years)	Mean (SD)	41.1 (15.6)	
	<18	309	1.1%
	18–30	8809	29.8%
	31–40	6632	22.4%
	41–50	4916	16.6%
	51–60	5176	17.5%
	61–70	2666	9.0%
	71–80	880	3.0%
	>80	171	0.6%
Sex	Female	14,174	48.0%
	Male	13,670	46.3%
	Unknown	1715	5.8%
Index year	2016	2932	9.9%
	2017	3367	11.4%
	2018	3860	13.1%
	2019	4401	14.9%
	2020	4818	16.3%
	2021	5359	18.1%
	2022	4822	16.3%
Biological drug	Adalimumab	10,911	36.9%
	Infliximab	9954	33.7%
	Vedolizumab	5846	19.8%
	Ustekinumab	2162	7.3%
	Golimumab	686	2.3%

Proportions of patients in N, % given, unless otherwise indicated. SD: standard deviation.

**Table 2:** Comparison of the number of patients with at least one prescription of each medication in the pre- and postindex periods.

	Twelve Months Prior to Index Date (N, %)	Twelve Months After Index Date (N, %)	*p*-Value ^1^
Corticosteroids	22,031 (74.5)	15,578 (52.7)	<0.001
5-ASA	15,746 (53.3)	10,786 (36.5)	<0.001
PPI	12,398 (41.9)	9233 (31.2)	<0.001
Analgesics	12,545 (42.4)	12,126 (41.0)	<0.001
Immunosuppressants	7945 (26.9)	3855 (13.0)	<0.001
Vitamin D	5791 (19.6)	5258 (18.1)	<0.001
Iron	4997 (16.9)	6314 (21.4)	<0.001
Antibiotics	8722 (29.5)	8545 (28.9)	<0.001
At least one drug	26,963 (91.2)	25,923 (87.7)	<0.001

^1^ McNemar test.

**Table 3:** Comparison of the number of prescribed pills per patient of each medication in the pre- and postindex periods.

	Twelve Months Prior to Index Date (Mean, SD)	Twelve Months After Index Date (Mean, SD)	Mean Difference (95% CI) ^1^	*p*-Value ^1^
Corticosteroids	183 (202)	105 (175)	−77.9 (−80.3–−75.5)	<0.001
5-ASA	242 (375)	180 (353)	−61.6 (−65.2–−58.1)	<0.001
PPI	68 (118)	63 (125)	−6.6 (−9.5–−3.7)	<0.001
Analgesics	36 (91)	40 (100)	2.4 (1.5–3.2)	<0.001
Immunosuppressants	104 (232)	49 (163)	−55.0 (−57.5–−52.6)	<0.001
Vitamin D	20 (53)	19 (53)	−1.5 (−2.1–−0.8)	<0.001
Iron	11 (40)	12 (42)	0.8 (0.3–1.3)	0.003
Antibiotics	7 (14)	7 (14)	−0.1 (−0.3–0.1)	0.291
Total pills *	704 (1712)	514 (1651)	−193.5 (−199.8–−187.2)	<0.001

^1^ Poisson regression with repeated measures adjusted for age and sex. * The number of total pills is the sum of the pill counts of the medications specified in the table.

**Table 4:** Comparison of the number of prescribed pills per patient of selected medications in the pre- and postindex periods stratified by age and sex.

		Twelve Months Prior to Index Date (Mean, SD)	Twelve Months After Index Date (Mean, SD)	Mean Difference (95% CI) ^1^	*p*-Value ^1^
Corticosteroids					
Age (years)	≤30	169 (183)	92 (159)	−76.5 (−80.7–−72.3)	<0.001
	31–40	174 (195)	100 (173)	−73.7 (−78.7–−68.7)	<0.001
	41–50	188 (209)	110 (179)	−77.6 (−83.4–−71.8)	<0.001
	51–60	196 (217)	113 (182)	−83.1 (−89.0–−77.2)	<0.001
	>60	213 (225)	129 (197)	−84.4 (−91.6–−77.3)	<0.001
Sex	Female	182 (202)	103 (172)	−78.7 (−82.0–−75.5)	<0.001
	Male	185 (204)	108 (179)	−77.0 (−80.5–−73.4)	<0.001
5-ASA					
Age (years)	≤30	200 (326)	137 (301)	−62.4 (−68.4–−56.5)	<0.001
	31–40	231 (361)	175 (346)	−56.0 (−63.3–−48.8)	<0.001
	41–50	256 (387)	193 (368)	−62.9 (−71.8–−53.9)	<0.001
	51–60	273 (409)	209 (384)	−63.5 (−72.3–−54.8)	<0.001
	>60	303 (426)	236 (401)	−66.6 (−77.6–−55.6)	<0.001
Sex	Female	233 (372)	171 (348)	−61.6 (−66.4–−56.9)	<0.001
	Male	253 (378)	190 (358)	−61.7 (−67.0–−56.5)	<0.001
Immunosuppressants					
Age (years)	≤30	103 (224)	51 (161)	−50.7 (−55.1–−46.3)	<0.001
	31–40	108 (237)	53 (166)	−54.9 (−60.3–−49.5)	<0.001
	41–50	116 (248)	52 (176)	−62.7 (−69.1–−56.3)	<0.001
	51–60	108 (240)	48 (166)	−59.6 (−65.6–−53.7)	<0.001
	>60	80 (203)	32 (137)	–48.0 (−54.1–−41.9)	<0.001
Sex	Female	95 (218)	43 (152)	−51.4 (−54.6–−48.2)	<0.001
	Male	115 (237)	55 (175)	−60.2 (−64.1–−56.3)	<0.001

^1^ The Poisson models with repeated measures stratified by age were adjusted by sex, while those stratified by sex were adjusted by age. The overall unstratified models for each medication were adjusted by both age and sex.

## Data Availability

The data and the code used for this study are available from the corresponding author upon reasonable request.
